# Relationship between *CYP7A1* -204A > C polymorphism with gallbladder stone disease and serum lipid levels: a meta-analysis

**DOI:** 10.1186/1476-511X-13-126

**Published:** 2014-08-08

**Authors:** Qiang Cai, Zhen-Qiang Wang, Qu Cai, Chen Li, Er-Zhen Chen, Zhao-Yan Jiang

**Affiliations:** Department of Surgery, Shanghai Institute of Digestive Surgery, Ruijin Hospital, Shanghai JiaoTong University School of Medicine, 200025 Shanghai, China; Department of Emergency, Ruijin Hospital, Shanghai JiaoTong University School of Medicine, 200025 Shanghai, China

**Keywords:** Gallbladder stone disease, Cholesterol 7α-hydroxylase, Serum lipids, Polymorphism, Meta-analysis

## Abstract

**Background:**

The *CYP7A1* gene polymorphism has been reported to be associated with gallbladder stone disease (GSD) and serum lipid levels, but the results were inconsistent. This meta-analysis aimed to evaluate the influence of the -204A > C polymorphism in the promoter of *CYP7A1* gene on the GSD and serum lipid levels.

**Methods:**

According to inclusion/exclusion criteria, eligible studies on *CYP7A1* gene -204A > C polymorphism of serum lipid levels and the risk of GSD were retrieved. Depending on the between-study heterogeneity, the fixed- or random-effects model was applied, and the data were analyzed using the RevMan software (V5.2).

**Results:**

Five studies totaling 830 GSD patients and 882 healthy controls were used to evaluate the relation of *CYP7A1* -204A > C polymorphism with GSD. Overall comparison of alleles A with C in all study population yielded 5% but non-significant increased risk of GSD (OR = 1.05, 95% CI: 0.91 − 1.22, P = 0.48). Subgroup analysis by ethnic differences did not show any association between *CYP7A1* -204A > C polymorphism and GSD either. Four studies totaling 802 cases and 691 controls were used to assess the relation of *CYP7A1* -204A > C polymorphism with serum lipid levels. All the subjects were from the Asian population. The pooled effects indicated that AC genotype had higher levels of TG than AA (MD = -0.42, 95% CI: -0.76 − -0.08, P = 0.01). CC genotype in cases had higher levels of TC (MD = 0.65, 95% CI: 0.25 − 1.05, P = 0.001) and LDL-C (MD = 0.40, 95% CI: 0.06 − 0.73, P = 0.02) than AA, AA (MD = -0.35, 95% CI: -0.60 − -0.10, P = 0.007) and AC (MD = −0.35, 95% CI: -0.61 − -0.08, P = 0.01) genotypes in controls had higher levels of TC than CC, and AA genotype in controls had higher levels of HDL-C than CC (MD = -0.15, 95% CI: -0.21 − -0.09, P < 0.00001).

**Conclusions:**

The *CYP7A1* -204A > C polymorphism is significantly associated with serum lipid levels in Asian population, but not gallbladder stone disease.

**Electronic supplementary material:**

The online version of this article (doi:10.1186/1476-511X-13-126) contains supplementary material, which is available to authorized users.

## Introduction

Gallbladder stone disease (GSD) is one of the most common diseases in many countries. The formation of GSD is multi-factorial, with a complex interaction between the environment factors and multiple susceptible genes [1]. Data from the Swedish Twin Registry showed that the contribution of hereditary factors to symptomatic GSD accounts for 25% [2].

Oversaturatation of biliary cholesterol is the requisite biochemical defect for the formation of GSD [3]. This pathophysiological change is induced by either hypersecretion of biliary cholesterol or decreased secretion of bile acids. Both the cholesterol secreted into bile and the bile acids converted from cholesterol in the liver are involved in the regulating cholesterol homeostasis. Cholesterol 7α-hydroxylase (CYP7A1) is the rate-limiting enzyme of hepatic bile acid synthesis. A rare mutation of CYP7A1 gene was reported to account for the incidence of gallstone disease as well as familial hypercholesterolemia in a family [4]. Various genetic variations have also been reported in *CYP7A1* gene [5, 6]. The polymorphism of -204A > C (rs3808607) in the promoter of CYP7A1 gene was reported to affect its enzyme activity [7]. A number of studies have been focused on the association between the -204A > C polymorphism and metabolic disorders of cholesterol and bile acid, including hypercholesterolemia, hypertriglyceridemia and GSD [8–13]. However, the results are inconsistent and inconclusive due to different study design, population, etc. Therefore, we performed this meta-analysis to evaluate the relation of *CYP7A1* -204A > C polymorphism with GSD as well as serum lipid levels.

## Materials and methods

### Search strategy

We conducted a systematic publication search that published in English or Chinese via pubic database PubMed, Embase, ISI Wed of Knowledge, China Biological Medicine (CBM) and China National Knowledge Infrastructure (CNKI) up to February 2014 using the following terms: “Cholesterol 7α-hydroxylase”, “CYP7A1”, “rs3808607 (-204A > C) polymorphism”, “gallbladder stone disease”, “dyslipidemia” and “serum lipid levels”. The search was restricted to humans. All eligible studies were retrieved and the full text of the articles was examined to make sure the data of interest were included. If multiple reports from the same patients were found, only the publication with the most complete data set was included.

### Inclusion and exclusion criteria

Studies that we identified were required to meet the following criteria: (1) study on -204A > C polymorphism of CYP7A1 gene, serum lipid levels and the risk of GSD; (2) case–control study that used either hospital-based or population-based designs; (3) reporting at least one relevant outcomes of association between genotype and serum lipid levels and the risk of GSD, serum lipid levels including total cholesterol (TC), high-density lipoprotein cholesterol (HDL-C), low-density lipoprotein cholesterol (LDL-C), and triglycerides (TG). Studies were excluded if they were case-only studies, case reports, or published abstracts from meeting.

### Extracted information

Two investigators (QC and ZQW) independently extracted the following information from all selected articles: first author, year, country, ethnicity, eligible subjects, study design, methods to diagnosis, genotyping information (genotyping method, number of genotypes, genotype distribution in cases and controls), association between genotypes and serum lipid parameters and the risk of GSD, etc. Ethnic backgrounds were categorized as Caucasian or Asian. The units of measurements used in this study were transformed into the standard measurements units.

### Statistical analysis

Before estimating the relationship between the *CYP7A1* -204A > C polymorphism and GSD and serum lipid levels, we tested whether the genotype frequencies of the controls were in Hardy-Weinberg equilibrium (HWE) using a χ^2^ test (P > 0.05) [14]. We carried out statistical analysis by the software Review Manager (V5.2) for Mac Os X. Continuous variables were expressed as mean difference (MD) with 95% confidence intervals (CI). Dichotomous variables were expressed as odd ratio (OR) with 95% CI. Subgroup analysis for ethinicity (Asian and Caucasian) and population (case and control) was conducted. The chi-square test based on *Q* test and *I*^*2*^ statistics were used to assess the hererogeneity among studies [15–17]. When the *Q* test was significant (P < 0.05) or *I*^*2*^ > 50%, indicating the presence of heterogeneity, a random-effects model (the DerSimonian & Laird method) was used [18]; otherwise, the fixed-effects model (the Mantel-Haenszel method) was used [19]. If enough studies were identified, funnel plots were to be used to investigate reporting biases.

## Results

### Studies and populations

**Figure 1:**
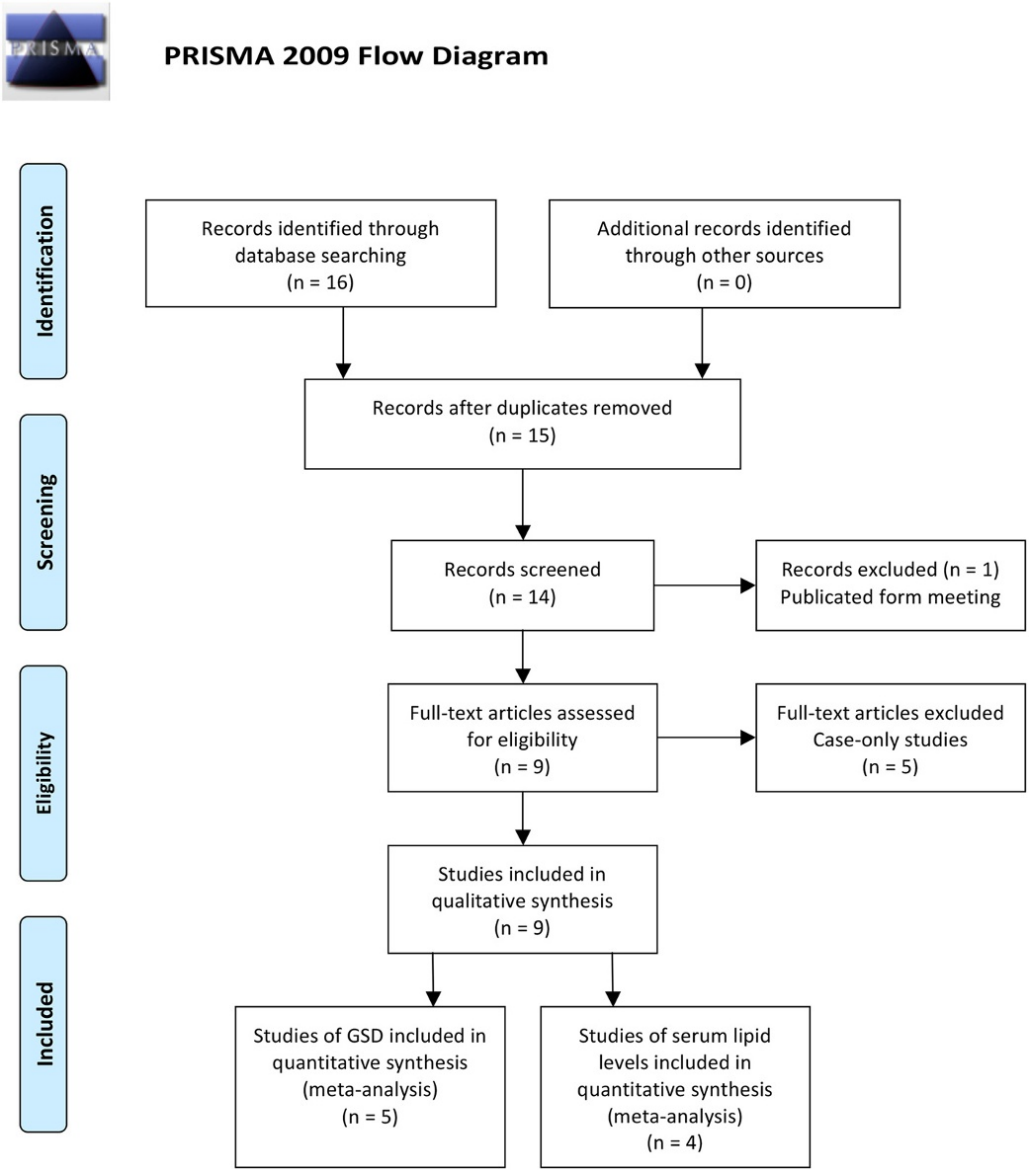
**Flow diagram of search strategy and study selection.**

The literature search identified 16 potentially relevant papers. Seven papers were excluded and 9 papers (5 in English [5, 11, 12, 20, 21], 4 in Chinese [22–25]) were included finally. A flow diagram of the study selection process is presented in Figure 1. All of the studies had been approved by the Ethics Committee of their affiliations, in accordance with the Helsinki Declaration of 1975 as revised in 1983, and all subjects had given informed consent. The present study was approved by the Ethics Committee of the Ruijin Hospital, Shanghai JiaoTong University School of Medicine. Five of the eligible studies including 830 gallbadder stone disease patients and 882 controls were used to evaluate the relation of *CYP7A1* -204A > C polymorphism with GSD [5, 11, 12, 21, 23]. Four of the eligible studies including 802 cases and 691 controls were used to assess the association between *CYP7A1* -204A > C polymorphism and serum lipid levels [20, 22, 24, 25]. Many studies have proved that serum lipid concentrations are strongly correlated to the risk of coronary artery disease (CAD), two papers contained the data of CAD was elected for the meta-analysis [20, 24]. The main characteristics of each study are presented in Additional file 1: Table S1.

### Analyses of the risk of GSD

**Figure 2:**
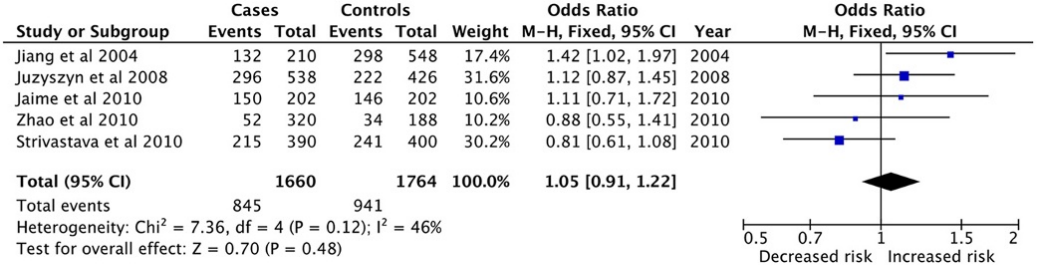
**Forest plot of the association between** ***CYP7A1*** **-204A > C polymorphism and GSD risk.** (Allelic model: A vs C).

In allelic model, the eligible compared groups were pooled with the fixed-effects models and the comparison showed that the *CYP7A1* -204A allele was related to a nonsignificant 5% increased risk of GSD (OR = 1.05, 95% CI: 0.91 − 1.22, P = 0.48) (Figure 2). No significance was observed in genotypic models for comparisons of AA (OR = 1.06, 95% CI: 0.79 − 1.42, P = 0.68) and AC (OR = 0.92, 95% CI: 0.70 − 1.21, P = 0.55) genotypes with CC genotype, respectively, as well as in dominant (OR = 0.97, 95% CI: 0.76 − 1.25, P = 0.82) and recessive (OR = 1.15, 95% CI: 0.92 − 1.43, P = 0.21) models (Table 1).

**Table 1:** **Comparisons of A vs C in allele, genotype dominant and recessive models for GSD risk**

Comparisons	Pooled OR (95% CI)	Z (P)	***I*** ^***2***^(%)
A vs C	1.05 (0.91,1.22)	0.70 (0.48)	46
AA vs CC	1.06 (0.79,1.42)	0.42 (0.68)	41
AC vs CC	0.92 (0.70,1.21)	0.60 (0.55)	0
AA + AC vs CC	0.97 (0.76,1.25)	0.22 (0.82)	12
AA vs AC + CC	1.15 (0.92,1.43)	1.25 (0.21)	31

Considering the ethnic differences might bias the overall association, we separated the studies in Asian and Caucasian. There was no significant change in all subgroups either (data not shown).

### Analyses of the serum lipid levels

**Figure 3:**
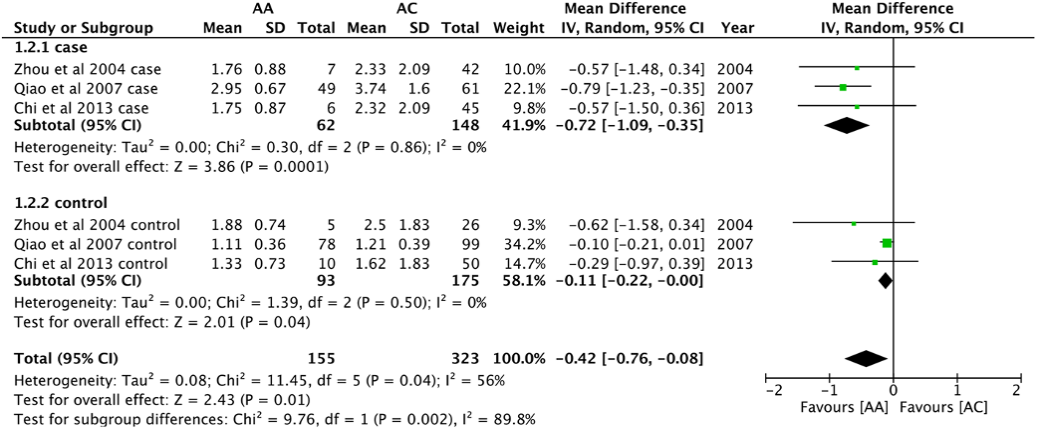
**Forest plot of the association between** ***CYP7A1*** **-204A > C polymorphism and serum TG levels.** (Genetic model: AA vs AC).

**Figure 4:**
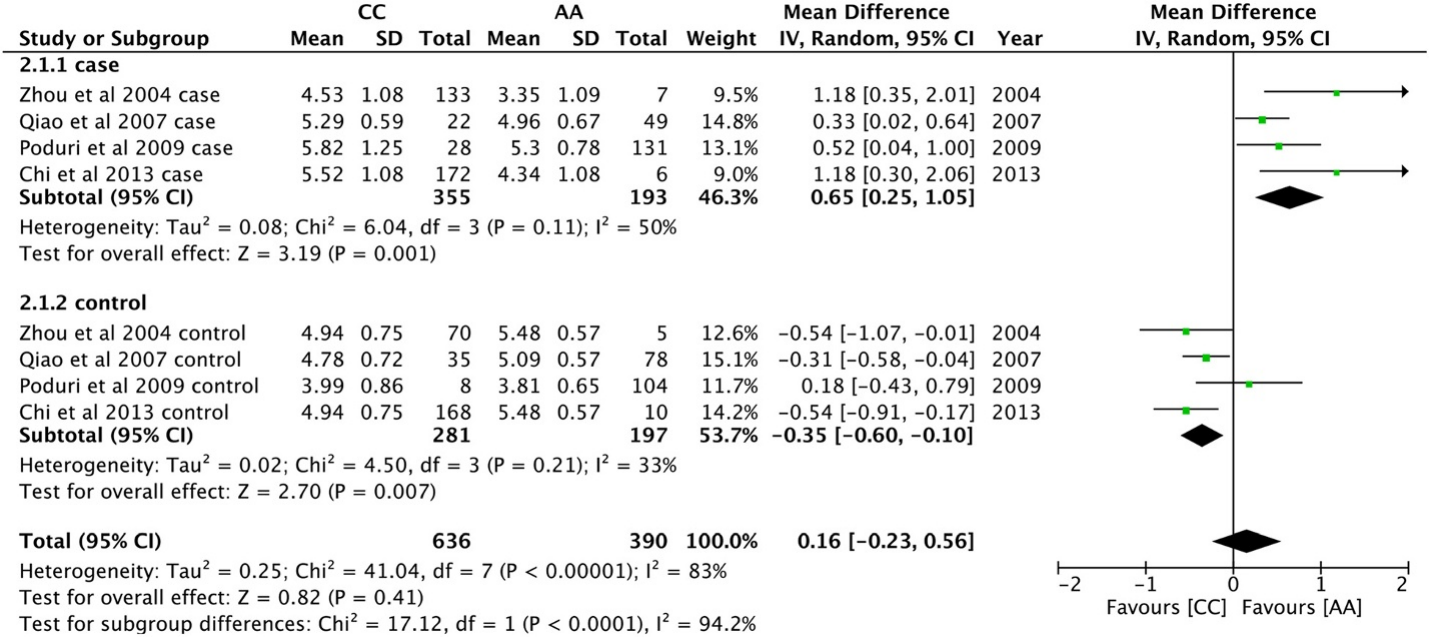
**Forest plot of the association between** ***CYP7A1*** **-204A > C polymorphism and serum TC levels.** (Genetic model: AA vs CC).

**Figure 5:**
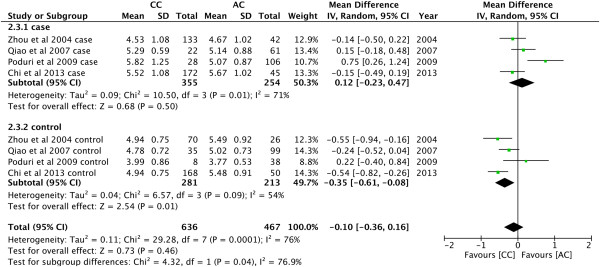
**Forest plot of the association between** ***CYP7A1*** **-204A > C polymorphism and serum TC levels.** (Genetic model: AC vs CC).

**Figure 6:**
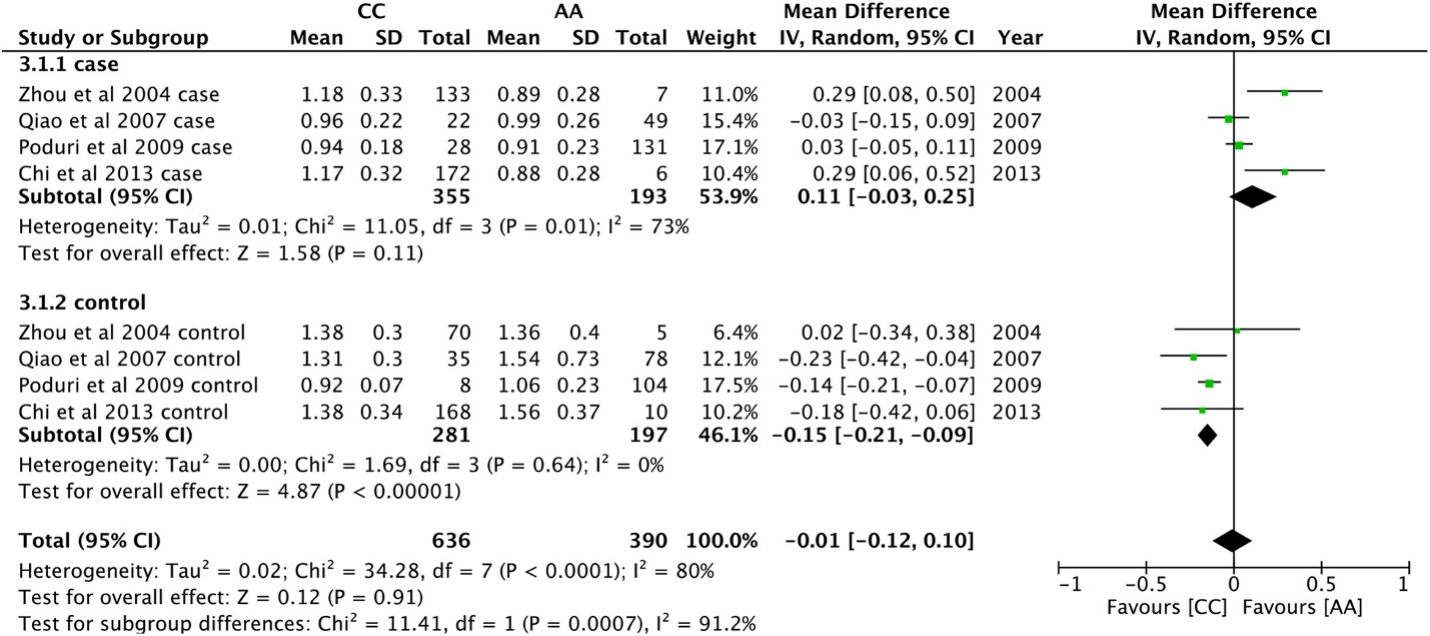
**Forest plot of the association between** ***CYP7A1*** **-204A > C polymorphism and serum HDL-C levels.** (Genetic model: AA vs CC).

**Figure 7:**
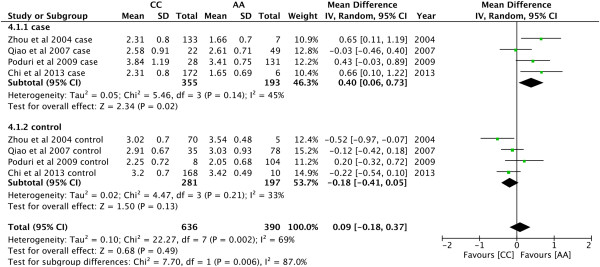
**Forest plot of the association between** ***CYP7A1*** **-204A > C polymorphism and serum LDL-C levels.** (Genetic model: AA vs CC).

In the present study, we included four studies in which all the subjects were Asian population. As shown in Figure 3, the pooled effects indicated that AC genotype had higher levels of TG than AA (MD = −0.42, 95% CI: -0.76 − -0.08, P = 0.01). Meanwhile, the magnitude of association was largely strengthened in cases (MD = -0.72, 95% CI: -1.09 − -0.35, P = 0.0001). As shown in Figure 4, AA genotype had higher levels of TC than CC in controls (MD = -0.35, 95% CI: -0.60 − -0.10, P = 0.007), but not in the recessive models in cases (MD = 0.65, 95% CI: 0.25 − 1.05, P = 0.001). AC genotype had higher levels of TC than CC in controls (MD = −0.35, 95% CI: -0.61 − -0.08, P = 0.01; Figure 5). There was no significant difference in the levels of TC between AA and AC genotypes (MD = −0.09, 95% CI: −0.31—0.13, P = 0.40). Compared with CC genotype in controls, AA had higher levels of HDL-C (MD = −0.15, 95% CI: -0.21 − -0.09, P < 0.00001; Figure 6). For the comparison of LDL-C levels, CC genotype was significantly higher than AA in cases (MD = 0.40, 95% CI: 0.06 − 0.73, P = 0.02; Figure 7).

### Test of heterogeneity

For the analyses of the serum lipid levels, the *I*^*2*^ values of heterogeneity were greater than 50% and P values of heterogeneity were less than 0.10 in all of the mentioned models above in the overall populations, indicating that significant between-study heterogeneity among the studies. In order to explore the possible sources of heterogeneity, we seperated the studies into cases and controls. Thereafter, the between-study heterogeneity was obviously reduced or even gone under the subgroup analyses.

## Discussion

To our knowledge, this is the first meta-analysis to evaluate the association between *CYP7A1* -204A > C polymorphism and GSD and serum lipid levels. In this study, we collected data from 9 papers to evaluate the association of CYP7A1 gene polymorphisms with GSD and serum lipid levels. The results showed that -204A > C polymorphism of CYP7A1 gene related with difference in serum lipids. However, this polymorphism was not associated with GSD.

The -204A > C of CYP7A1 gene is one of the most frequently studied polymorphisms for the association with GSD. Our previous study showed that A allele of CYP7A1 gene might be considered as risk gene for GSD in Chinese patients [21]. Later on, Juzyszyn et al. [11] and Strivastava et al. [5], using larger samples from Polish and Indian, did not confirm such association. The samples sizes in the rest studies were relatively small [12, 23]. Herein, by pooling all the previous studies, we demonstrated a lack of association between this polymorphism with GSD.

An obvious difference of gallstone prevalence between populations is present due to different ethnicities. GSD is highly prevalent in Pima Indians, Hispanic, relatively lower in Asian and the lowest in African [26]. The frequency of A allele of -204A > C polymorphism in gallstone-free subjects is lower in Asian population, 18.09% in Chinese [23], high up to 60.2% in Indian [5]. In Caucasian population, its frequency is between 52.1 [11] and 72.28% [12]. However, when the population was divided into Asian and Caucasian, we did not find any association of -204A > C polymorphism with GSD existed in either ethnicity.

The second aim of our study is to evaluate the association between -204A > C polymorphism and serum lipids. The -204A > C polymorphism was shown to be associated with plasma LDL-C concentrations [8, 27]. Our previous study also found that individuals with A allele tended to have lower LDL-C concentrations [21]. While in this meta-analysis, we found that the genotype AA had significantly lower levels of LDL-C than genotype CC only in patients, but not in controls. Couture et al. [8] described that the genotype AC had significantly higher TG levels than the genotype CC in women and the C variant was also associated with an increased TC/HDL-C ratio in men. Hofman et al. [10] found a significant 34% increase of serum TG levels in genotype AA as compared with genotype CC in a healthy normolipidaemic male population. However, our meta-analysis showed that genotype AC had higher TG levels than genotype AA, allele A carriers in healthy population had higher TC levels than genotype CC, but not in the recessive models in cases, and genotype AA had higher HDL-C levels than genotype CC in controls.

Some limitations of this meta-analysis merit serious consideration. First, only the papers published in English and Chinese were included in our study. Any data reported in other languages could not be included which might bring some bias. Second, no adequate information such as source of the subjects, anti-dyslipidemia drug and etc. could be obtained in this meta-analysis. These factors might bring in several possible sources of heterogeneity. Third, most studies have recruited age > 40 years, for whom environmental factors are likely to contribute more prominently than the genetic component during the development of GSD and dyslipidemia.

In conclusion, this meta-analysis did not found any association between *CYP7A1* -204A > C polymorphism and GSD. However, this polymorphism was closely related with serum lipid levels.

## Electronic supplementary material

Additional file 1: Table S1: Supporting Information. (XLS 56 KB)
